# The prognostic value of pulmonary embolism severity index in acute pulmonary embolism: a meta-analysis

**DOI:** 10.1186/1465-9921-13-111

**Published:** 2012-12-04

**Authors:** Xiao-Yu Zhou, Su-Qin Ben, Hong-Lin Chen, Song-Shi Ni

**Affiliations:** 1 Department of Respiratory Diseases, the Affiliated Hospital of Nantong University, Xi Si Road 20#, Nantong City, Jiangsu Province, 226001, People’s Republic of China; 2 Nantong University, Qi Xiu Road 19#, Nantong City, Jiangsu Province, 226001, People’s Republic of China

**Keywords:** Acute pulmonary embolism, Pulmonary embolism severity index, Prognosis

## Abstract

**Background:**

Prognostic assessment is important for the management of patients with acute pulmonary embolism (APE). Pulmonary Embolism Severity Index (PESI) and simple PESI (sPESI) are new emerged prognostic assessment tools for APE. The aim of this meta-analysis is to assess the accuracy of the PESI and the sPESI to predict prognostic outcomes (all-cause and PE-related mortality, serious adverse events) in APE patients, and compare between these two PESIs.

**Methods:**

MEDLINE and EMBASE database were searched up to June 2012 using the terms “Pulmonary Embolism Severity Index” and “pulmonary embolism”. Summary odds ratio (OR) with 95% confidence intervals (CIs) for prognostic outcomes in low risk PESI versus high risk PESI were calculated. Summary receiver operating characteristic curve (SROC) used to estimate overall predicting accuracies of prognostic outcomes.

**Results:**

Twenty-one studies were included in this meta-analysis. The results showed low-risk PESI was significantly associated with lower all-cause mortality (OR 0.13; 95% CI 0.12 to 0.15), PE-related mortality (OR 0.09; 95% CI 0.05 to 0.17) and serious adverse events (OR 0.34; 95% CI 0.29 to 0.41), with no homogeneity across studies. In sPESI subgroup, the OR of all-cause mortality, PE-related mortality, and serious adverse events was 0.10 (95% CI 0.08 to 0.14), 0.09 (95% CI 0.03 to 0.26) and 0.40 (95% CI 0.31 to 0.51), respectively; while in PESI subgroup, the OR was 0.14 (95% CI 0.13 to 0.16), 0.09 (95% CI 0.04 to 0.21), and 0.30 (95% CI 0.23 to 0.38), respectively. For accuracy analysis, the pooled sensitivity, the pooled specificity, and the overall weighted AUC for PESI predicting all-cause mortality was 0.909 (95% CI: 0.900 to 0.916), 0.411 (95% CI: 0.407 to 0.415), and 0.7853±0.0058, respectively; for PE-related mortality, it was 0.953 (95% CI: 0.913 to 0.978), 0.374 (95% CI: 0.360 to 0.388), and 0.8218±0.0349, respectively; for serious adverse events, it was 0.821 (95% CI: 0.795 to 0.845), 0.389 (95% CI: 0.384 to 0.394), and 0.6809±0.0208, respectively. In sPESI subgroup, the AUC for predicting all-cause mortality, PE-related mortality, and serious adverse events was 0.7920±0.0117, 0.8317±0.0547, and 0.6454±0.0197, respectively. In PESI subgroup, the AUC was 0.7856±0.0075, 0.8158±0.0451, and 0.6609±0.0252, respectively.

**Conclusions:**

PESI has discriminative power to predict the short-term death and adverse outcome events in patients with acute pulmonary embolism, the PESI and the sPESI have similar accuracy, while sPESI is easier to use. However, the calibration for predicting prognosis can’t be calculated from this meta-analysis, some prospective studies for accessing PESI predicting calibration can be recommended.

## Background

Epidemiological studies showed the prevalence of acute pulmonary embolism (APE) among hospitalized patients in the United States, according to data collected between 1979 and 1999, was 0.4% [1]. In Europe, a total of 67,351(in 2005), 69,234 (in 2006) and 71,223(in 2007) APE were coded either as principal or secondary diagnosis in German hospitals according to the data from Federal Statistical Office [2]. In Asia, from a multi-center registration study of Chinese hospitals between 1997 and 2008, a total of 18,206 patients were confirmed with APE from 16,972,182 hospital admissions, the annual incidence was 0.1% (95% CI: 0.1% to 0.2%) [3]; while in Korean, incidence of established APE was 88 (0.17%) of 50,882 identified retrospectively from patients hospitalized during a 2-year period from 2005 to 2007[4]. APE has become a relatively common cardiovascular emergency.

The key consequences of a pulmonary thromboembolic episode are haemodynamic. Large and multiple emboli might abruptly increase pulmonary vascular resistance to a level of after load which cannot be matched by the right ventricle (RV), and sudden death may occur [5]. In the ICOPER registry, the 14-day mortality of APE was above 20% [6]. In the RIETE registry, at 3 months, the cumulative rates of overall mortality and fatal PE were 8.65% and 1.68%, respectively [7]. While in the EMPEROR registry, the all-cause 30-day mortality rate was 5.4% (95% CI: 4.4% to 6.6%) [8]. APE has become one of the leading causes of preventable hospital deaths.

The mortality of APE can be predicted by haemodynamic status, right ventricular dysfunction, myocardial injury, and other clinical and routine laboratory tests. Shock and hypotension are principal markers of high risk of early death in APE. RV dysfunction which is detected by echocardiography, computed tomography, and brain natriuretic peptide (BNP) is related to intermediate risk of short-term mortality in APE. Myocardial injury in patients with APE is related to an intermediate risk of short-term mortality, which can be detected by troponin T or I testing [5]. While the Pulmonary Embolism Severity Index (PESI) was recently found to be a well validated and highly reliable clinical prognostic model for patients with APE.

The PESI was designed by Aujesky D and his colleague in 2005, which comprises 11 routinely available clinical predictor variables with different prognostic weights [9]. On the basis of the PESI score, each patient is classified into one of five classes (I-V), with 30-day mortality ranging from 1.1% to 24.5%. Patients in risk classes I and II are categorized as low-risk and in risk classes III-V are categorized as high-risk. While the PESI is hard to memorizes for based on 11 items, and it’s difficult to calculate in busy emergency departments. The simplified PESI (sPESI) was developed in 2010, which includes six of the 11 original PESI variables [10]. Patients with none of the variables (0 points) are categorized as low-risk; with one to six the variables (1–6 points) are categorized as high-risk. The PESI and sPESI are listed in the Additional file 1: Appendix 1.

The prognostic value of the PESI and sPESI has been assessed by some studies, but has not been systematic reviewed. We conduct this meta-analysis to assess the accuracy of the PESI and the sPESI to predict prognostic outcomes in APE patients. In particular, we also compared the predicting accuracy between these two PESIs.

## Materials and methods

### Eligibility criteria

We defined studies as being eligible for inclusion in this analysis if they met the following criteria: (1) types of studies: observational studies ( cohort studies and case–control studies ) were included; (2) types of participants: population of objective diagnosis of APE with short-term prognosis outcome were included; (3) types of outcomes: studies included at least one of three prognostic outcomes: all-cause mortality, PE-related mortality, serious adverse events; and a 2×2 table of outcome results could be constructed based on low-risk PESIs or high-risk PESIs.

### Search strategy and study selection

We searched MEDLINE (PubMed, http://www.ncbi.nlm.nih.gov/pubmed/), and EMBASE (http://www.embase.com) up to June 2012. The search strategy included terms of “Pulmonary Embolism Severity Index” and “Pulmonary Embolism”. The search was limited to English- language articles. We did not appoint limit in country, race, or publication year. To identify any additional relevant studies, we also hand-searched conference proceedings and scanned references of retrieved articles.

Study selection was initially performed by review of titles and abstracts. When there was any possibility that it might be relevant, the full text were downloaded and then reviewed for data retrieval.

Two reviewers independently judged study eligibility while screening the citations. Disagreements were resolved by a third reviewer.

### Quality assessment

We used Newcastle–Ottawa Scale (NOS) to assess the quality of the included observational studies [11]. The NOS contains eight items, categorized into three dimensions including Selection (4), Comparability (1), and Exposure (3). A high quality study can be awarded a maximum of one star for each numbered item within the Selection and Exposure categories. A maximum of two stars can be given for Comparability. The NOS ranges between zero up to nine stars. The NOS is listed in the Additional file 2: Appendix 2.

Two reviewers independent assessed the quality of studies. Disagreements were resolved by a third reviewer.

### Data extraction

We collected information about study characteristics (study year, country, study design, number of patients, mean age, gender distribution, type of PESI, methods for diagnosis of APE, haemodynamic status, length of follow-up) and number of patients with the available outcomes of all-cause death, PE-related death, serious adverse events among low-risk or high-risk PESIs.

In this meta-analysis, all-cause mortality was considered as the primary outcome, PE-related death, serious adverse events were considered as the secondary outcome. Serious adverse events were defined as any of the following: nonfatal recurrent PE, nonfatal recurrent DVT, nonfatal bleeding, or delayed homodynamic instability.

Data were extracted independently by two reviewers. Any differences of opinion were resolved by discussion and consensus reached by discussion with a third reviewer.

### Statistical analysis

First, we considered high-risk PESI as a risk factor for APE prognostic adverse outcomes. Summary OR with 95% CIs for three prognostic outcomes in low-risk PESI versus high-risk PESI was calculated respectively. Statistical heterogeneity was explored by χ^2^ and inconsistency (I^2^) statistics; an I^2^ value of 50 percent or more represented heterogeneity [12]. If there was no heterogeneity, fixed effects model was used for meta-analysis; otherwise, a random effect model based on the DerSimonian and Laird estimator was used [13]. Overall effects were determined using the Z test. Potential publication bias was evaluated by the funnel plot. Subgroup meta-analyses were performed by PESI subgroup and sPESI subgroup. Sensitivity analysis was performed by only pooled prospective studies. The first step meta-analysis was performed with Review Manager (RevMan) software (version 5.0.21; Update Software Ltd, Oxford, Oxon, UK).

Second, we assess the accuracy (discriminative power) of PESI to predict APE prognostic outcomes. If we predict low-risk PESI as good prognostic outcomes, high-risk PESI as worse prognostic outcomes, the sensitivity (true positive rate) and specificity (true negative rate) of each included study was calculated compared to actual prognostic outcomes (the so-called gold standard). And the overall pooled sensitivity and specificity with 95% CIs was estimated by DerSimonian and Laird’s random-effects model [13]. In addition, summary receiver operator characteristic (SROC) analysis was performed to examine the interaction between sensitivity and (1-specificity) [14], and to quantify test performance using the area under the curve (AUC) and Q* value[15]. The second step meta-analyses were performed with Meta DiSc 1.4 (version 0.6; By Joseph Lau) [16].

## Results

### Description of included studies

**Figure 1:**
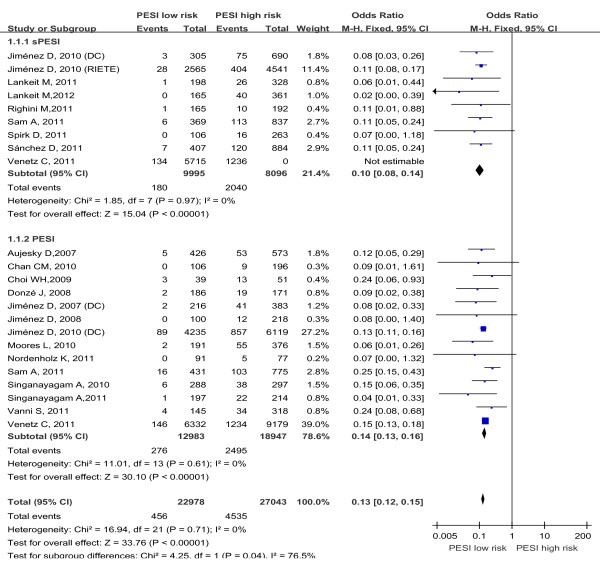
Flow diagram showing selection of studies.

Overall, 21 studies [10,17-36] were selected for this meta-analysis described in Figure 1. Among those studies, 10 studies were prospective cohorts, 9 studies were retrospective cohorts or case–control studies, the remaining 2 studies didn’t mention about the study design type. Eleven studies were multi-center studies. Countries involved in these studies were Germany, Spain, Poland, Greece, Switzerland, USA, Belgium, Italy, Greece, UK, Israel, and Korea. Most of the studies (20 studies) came from Europe and North America, only a study came form Asia. All of the 21 included studies used the same diagnostic criteria of PE, which included high clinical probability of PE, and confirmed by contrast-enhanced multi detector CT or ventilation- perfusion lung scan, or confirmed lower limb deep vein thrombosis (DVT) by venous ultrasound. A total of 5 studies included haemodynamic stable patients, a study included haemodynamic stable and unstable patients, but most of the studies didn’t mention about the haemodynamic status of the included patients. The length of follow-up ranged from hospital discharge to 6 month following admission to hospital. The NOS for methodological quality assessment of these 21 studies all scored at 7 of 8 star, indicating good quality. The main characteristics of the selected studies are reported in Table 1.

**Table 1:** Characteristics of Selected Studies

**First author, Year**	**Country**	**Study design**	**Patients (n)**	**Age (Year)**	**Gender (male/female)**	**Haemodynamic**	**Follow-Up**	NOS (*)
Lankeit M, 2011	Germany, Spain, Poland, Greece	Prospective	526	71 (55–79)	266/260	Stable	6 month	8
Sánchez D, 2011	Spain, Switzerland, USA	Retrospective	1291	74 (61–80)	579/712	Stable and unstable	30 day	7
Lankeit M,2012	Germany, Spain, Greece	Prospective	526	74 (61–80)	227/299	Stable and unstable	30 day	7
Righini M,2011	Switzerland, France, Belgium	Prospective	357	mean age 64	157/200	Stable and unstable	3 month	8
Spirk D, 2011	Switzerland	Prospective	369	20.9% >80	195/174	Stable and unstable	30 day	8
Vanni S, 2011	Italy	Prospective	463	67% > 65	208/254	Stable and unstable	in-hospital stay	8
Venetz C, 2011	Switzerland, Spain	Not mentioned	15531	67 (52–77)	6227/9304	Stable and unstable	30 day	7
Hariharan P, 2011	USA	Retrospective	245	57 ± 17	115/130	Stable and unstable	in-hospital stay	8
Jiménez D, 2011	Spain, Switzerland, USA, Greece	Not mentioned	591	74 (65–82)	254/337	Stable	30 day	7
Singanayagam A,2011	UK	Retrospective	411	55.4% >65	177/234	Stable and unstable	30 day	8
Jiménez D, 2010 (Derivation Cohort)	Spanish, France, Italy, Israel	Retrospective	10354	52.8% >65	4100/6254	Stable and unstable	30 day	7
Jiménez D, 2010 (Derivation Cohort)	Spanish, France, Italy, Israel	Retrospective	995	67.4% >65	449/546	Stable and unstable	30 day	7
Jiménez D, 2010 (RIETE Cohort)	Spanish, France, Italy, Israel	Retrospective	7106	68.2% >65	3232/3874	Stable and unstable	30 day	7
Sam A, 2011	Spain, USA, Switzerland	Prospective	1206	69.3±16.1	536/670	Stable and unstable	30 day	8
Chan CM, 2010	USA	Retrospective	302	59.7±17.2	133/169	Stable and unstable	90 day	8
Singanayagam A, 2010	UK	Retrospective	585	55.4% >65	258/327	Stable and unstable	30 day	7
Moores L, 2010	USA, Switzerland, Spain	Retrospective	567	74% >65	245/322	Stable	30 day	7
Nordenholz K, 2011	USA	Retrospective	168	53 (40–66)	72/96	Stable and unstable	30 day	7
Choi WH,2009	Korea	Retrospective	90	60.4±16.0	37/53	Stable and unstable	30 day	7
Donzé J, 2008	Switzerland, France, USA	Prospective	357	64±18	158/199	Stable	90 day	8
Jiménez D, 2008	Spain, USA	Prospective	318	75.5% >65	136/182	Stable	30 day	8
Jiménez D, 2007 (Validation Cohort)	Spain	Prospective	599	67% >65	246/353	Stable and unstable	30 day	8
Aujesky D,2007	Switzerland, France, USA	Prospective	899	64% >65	382/517	Stable and unstable	30 day	8

### Meta-analysis

#### High-risk PESI as a risk factor for adverse outcomes

**Figure 2:**
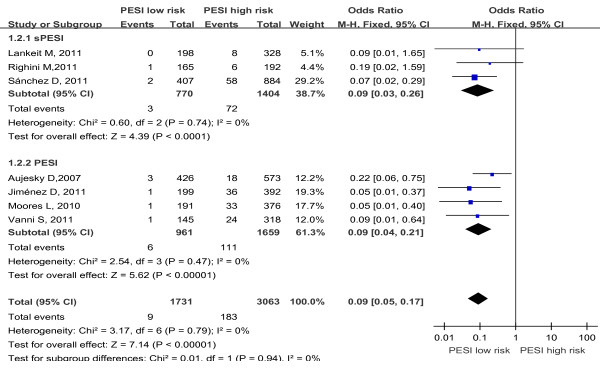
Meta-analysis of all-cause mortality in PE with PESI low risk versus PESI high risk.

**Figure 3:**
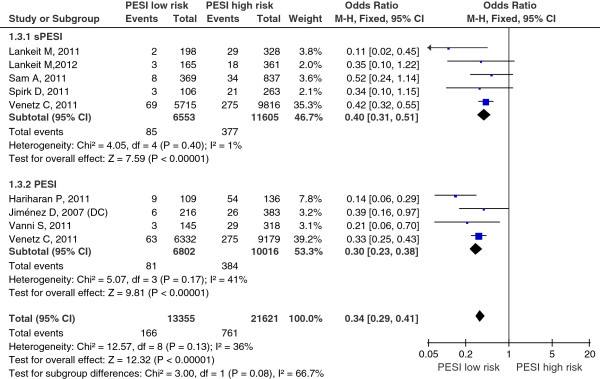
Funnel plots for publication bias analysis. OR= odds ratio; S.E.=standard error.

The association between PESI and PE adverse prognosis outcome risk of every included study are shown in Table 2. Data on all-cause mortality were reported in 19 studies. Five studies assessed the sPESI, 11 studies assessed the PESI, 3 studies assessed sPESI and PESI in the same study. A total of 50,021 patients were analyzed in the studies, including 4,991 patients with all-cause death. There was no heterogeneity in the studies (I^2^ = 0%). The pooled all-cause mortality was 2.0% (456/22978) in patients with low-risk PESI Vs 16.7% (4535/27043) in patients with high-risk PESI. The summary OR of patients with low-risk PESI compared with patients with high-risk PESI was 0.13 (95% CI 0.12 to 0.15; Z=33.76, P < 0.00001) (Figure 2). The funnel plot showed no asymmetry, which indicated no evidence of publication bias (Figure 3A). While in subgroup analysis for sPESI and PESI, the pooled all-cause mortality was 1.8% (180/9995) in patients with low-risk sPESI Vs 25.2%(2040/8096) in patients with high-risk sPESI, the summary OR of patients with low-risk sPESI compared with patients with high-risk sPESI was 0.10 (95% CI 0.08 to 0.14; Z=15.04, P < 0.00001) (Figure 2 Above); the pooled all-cause mortality was 2.1% (276/12983) in patients with low-risk PESI Vs 13.2% (2495/18947) in patients with high-risk PESI, the summary OR of patients with low-risk PESI compared with patients with high-risk PESI was 0.14 (95% CI 0.13 to 0.16; Z=30.10, P < 0.00001) (Figure 2 Below).

**Table 2:** PESI for PE risk and diagnosis

**First author, Year**	**PESI**	**Risk**						**Diagnosis**					
		**All-cause death**		**PE-related death**		**Adverse outcome**		**All-cause death**		**PE-related death**		**Adverse outcome**	
		**PESI low risk**	**PESI high risk**	**PESI low risk**	**PESI high risk**	**PESI low risk**	**PESI high risk**	**Sensitivity**	**Specific**	**Sensitivity**	**Specific**	**Sensitivity**	**Specific**
Lankeit M, 2011	sPESI	1/198 (0.5%)	26/328 (7.9%)	0/198 (0.0%)	8/328 (2.4%)	2/198 (1.0%)	29/328 (8.8%)	0.963	0.395	1.000	0.382	0.935	0.396
Sánchez D, 2011	sPESI	7/407 (1.7%)	120/884 (13.6%)	2/407 (0.5%)	58/884 (6.6%)	Not mentioned	Not mentioned	0.945	0.344	0.967	0.329	N/A	N/A
Lankeit M, 2012	sPESI	0/165 (0.0%)	40/361 (11.1%)	Not mentioned	Not mentioned	3/165 (1.8%)	18/361 (5.0%)	1.000	0.340	N/A	N/A	0.857	0.321
Righini M, 2011	sPESI	1/165 (0.9%)	10/192 (5.2%)	1/165 (0.9%)	6/192 (3.0%)	Not mentioned	Not mentioned	0.909	0.474	0.857	0.469	N/A	N/A
Spirk D, 2011	sPESI	0/106 (0.0%)	16/263 (6.1%)	Not mentioned	Not mentioned	3/106 (2.9%)	21/263 (8.0%)	1.000	0.394	N/A	N/A	0.875	0.299
Vanni S, 2011	PESI	4/145 (2.8%)	34/318 (10.7%)	1/145 (0.7%)	24/318 (7.5%)	3/145 (2.1%)	29/318 (9.1%)	0.895	0.332	0.960	0.329	0.906	0.329
Venetz C, 2011	PESI	146/6332 (2.3%)	1234/9179 (14.1%)	Not mentioned	Not mentioned	63/6332 (1.0%)	275/9179 (3.0%)	0.894	0.438	N/A	N/A	0.814	0.417
Venetz C, 2011	sPESI	134/5715 (2.7%)	1236/9816 (13.1%)	Not mentioned	Not mentioned	69/5715 (1.2%)	275/9816 (2.8%)	0.902	0.394	N/A	N/A	0.799	0.372
Hariharan P, 2011	PESI	Not mentioned	Not mentioned	Not mentioned	Not mentioned	9/109 (8.3%)	54/136 (39.7%)	N/A	N/A	N/A	N/A	0.857	0.549
Jiménez D, 2011	PESI	Not mentioned	Not mentioned	1/199 (0.5%)	36/392 (9.2%)	Not mentioned	Not mentioned	N/A	N/A	0.973	0.357	N/A	N/A
Singanayagam A, 2011	PESI	1/197 (0.5%)	22/214 (10.3%)	Not mentioned	Not mentioned	Not mentioned	Not mentioned	0.957	0.505	N/A	N/A	N/A	N/A
Jiménez D, 2010 (Derivation Cohort)	PESI	89/4235 (2.1%)	857/6119 (14.0%)	Not mentioned	Not mentioned	Not mentioned	Not mentioned	0.906	0.411	N/A	N/A	N/A	N/A
Jiménez D, 2010 (Derivation Cohort)	sPESI	3/305 (1.0%)	75/690 (10.9%)	Not mentioned	Not mentioned	Not mentioned	Not mentioned	0.962	0.329	N/A	N/A	N/A	N/A
Jiménez D, 2010 (RIETE Cohort)	sPESI	28/2565 (1.1%)	404/4541 (8.9%)	Not mentioned	Not mentioned	Not mentioned	Not mentioned	0.935	0.380	N/A	N/A	N/A	N/A
Sam A, 2011	PESI	16/431 (3.7%)	103/775 (13.3%)	Not mentioned	Not mentioned	Not mentioned	Not mentioned	0.866	0.382	N/A	N/A	N/A	N/A
Sam A, 2011	sPESI	6/369 (1.6%)	113/837 (13.5%)	Not mentioned	Not mentioned	8/369 (2.2%)	34/837 (4.1%)	0.950	0.334	N/A	N/A	0.810	0.310
Chan CM, 2010	PESI	0/106 (0.0%)	9/196 (4.6%)	Not mentioned	Not mentioned	Not mentioned	Not mentioned	1.000	0.362	N/A	N/A	N/A	N/A
Singanayagam A, 2010	PESI	6/288 (2.1%)	38/297 (12.8%)	Not mentioned	Not mentioned	Not mentioned	Not mentioned	0.864	0.521	N/A	N/A	N/A	N/A
Moores L, 2010	PESI	2/191 (1.0%)	55/376 (14.6%)	1/191 (1.5%)	33/376 (8.8%)	Not mentioned	Not mentioned	0.965	0.371	0.971	0.356	N/A	N/A
Nordenholz K, 2011	PESI	0/91 (0.0%)	5/77 (6.5%)	Not mentioned	Not mentioned	Not mentioned	Not mentioned	1.000	0.588	N/A	N/A	N/A	N/A
Choi WH, 2009	PESI	3/39 (7.7%)	13/51 (25.5%)	Not mentioned	Not mentioned	Not mentioned	Not mentioned	0.813	0.486	N/A	N/A	N/A	N/A
Donzé J, 2008	PESI	2/186 (1.1%)	19/171 (11.1%)	Not mentioned	Not mentioned	Not mentioned	Not mentioned	0.905	0.548	N/A	N/A	N/A	N/A
Jiménez D, 2008	PESI	0/100 (0.0%)	12/218 (5.5%)	Not mentioned	Not mentioned	Not mentioned	Not mentioned	1.000	0.327	N/A	N/A	N/A	N/A
Jiménez D, 2007 (Validation Cohort)	PESI	2/216 (0.9%)	41/383 (10.7%)	Not mentioned	Not mentioned	6/216 (2.8%)	26/383 (6.8%)	0.953	0.385	N/A	N/A	0.813	0.370
Aujesky D, 2007	PESI	5/426 (1.2%)	53/573 (9.2%)	3/426 (0.7%)	18/573 (3.1%)	Not mentioned	Not mentioned	0.914	0.447	0.857	0.433	N/A	N/A

Adverse outcome include nonfatal recurrent PE, nonfatal recurrent DVT, nonfatal bleeding, or delayed hemodynamic instability.

**Figure 4:**
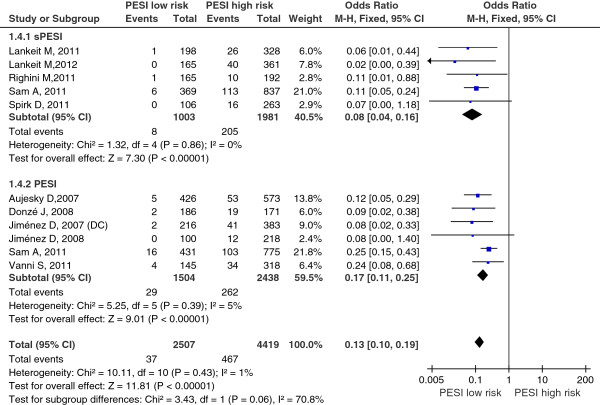
Meta-analysis of PE-related mortality in PE with PESI low risk versus PESI high risk.

Data on PE-related mortality were reported in 7 studies, 3 studies assessed the sPESI, and 4 studies assessed the PESI. A total of 4,794 patients were analyzed in the studies, including 192 patients with PE-related death. There was no heterogeneity in the studies (I^2^ = 0%). The pooled PE-related mortality was 0.5% (9/1731) in patients with low-risk PESI Vs 6.0% (183/3063) in patients with high-risk PESI. The summary OR of patients with low-risk PESI compared with patients with high-risk PESI was 0.09 (95% CI 0.05 to 0.17; Z=7.14, P < 0.00001) (Figure 4). The funnel plot showed no asymmetry, which indicated no evidence of publication bias (Figure 3B). While in subgroup analysis for sPESI and PESI, the pooled PE-related mortality was 0.4% (3/770) in patients with low-risk sPESI Vs 5.1% (72/1404) in patients with high-risk sPESI, the summary OR of patients with low-risk sPESI compared with patients with high-risk sPESI was 0.09 (95% CI 0.03 to 0.26; Z=4.39, P < 0.00001) (Figure 4 Above); the pooled PE-related mortality was 0.6% (6/961) in patients with low-risk PESI Vs 6.7% (111/1659) in patients with high-risk PESI, the summary OR of patients with low-risk PESI compared with patients with high-risk PESI was 0.09 (95% CI 0.04 to 0.21; Z=5.62, P < 0.00001) (Figure 4 Below).

**Figure 5:**
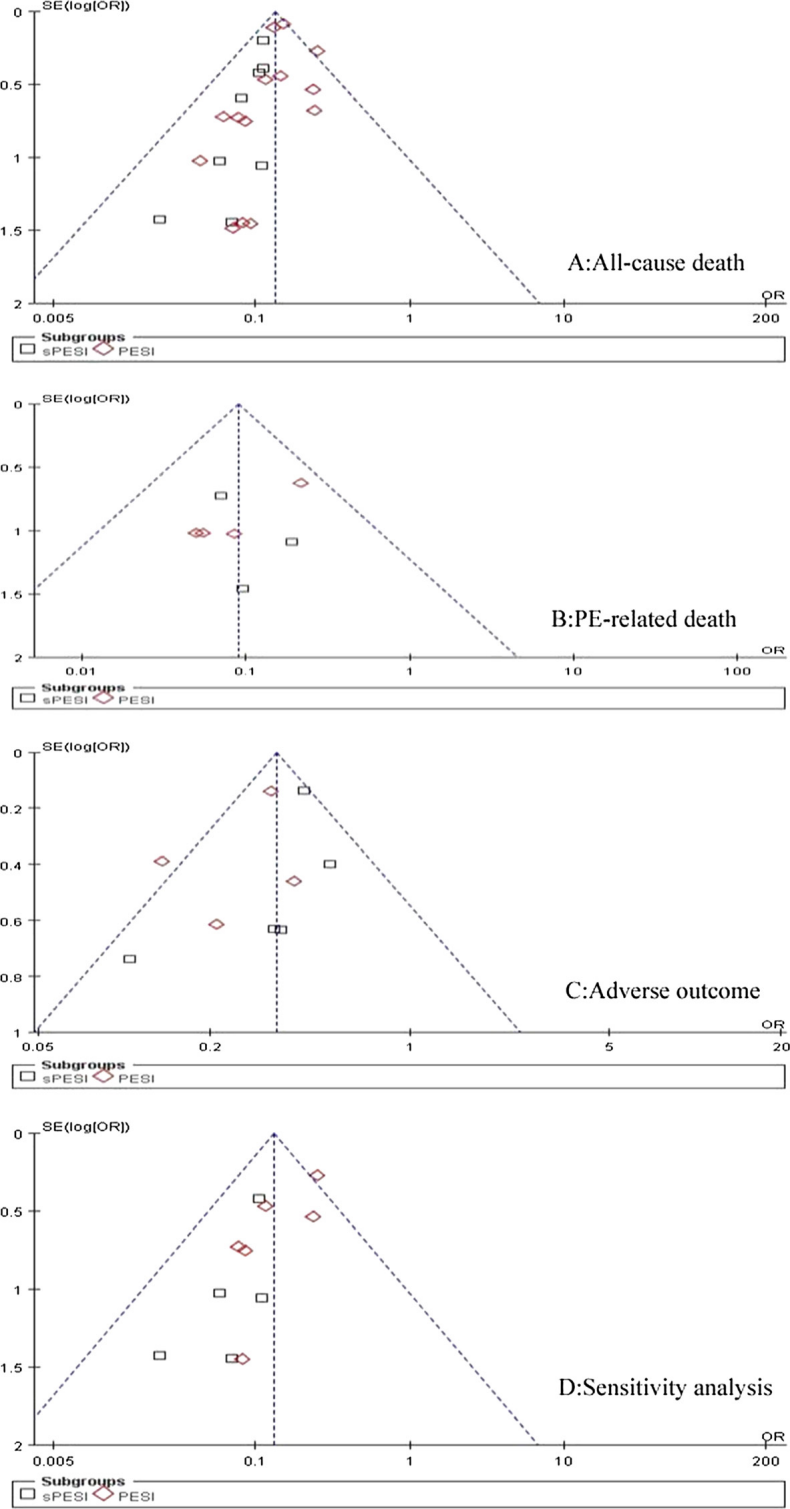
**Meta-analysis of serious adverse events in PE with PESI low risk versus PESI high risk.** Serious adverse events were defined as any of the following: nonfatal recurrent PE, nonfatal recurrent DVT, nonfatal bleeding, or delayed homodynamic instability.

Data on serious adverse events were reported in 8 studies, 4 studies assessed the sPESI, 3 studies assessed the PESI, a study assessed sPESI and PESI in the same study. A total of 50,021 patients were analyzed in the studies, including 4,991 patients with serious adverse events. There was no heterogeneity in the studies (I^2^ = 36%). The pooled serious adverse events rate was 1.2% (166/13355) in patients with low-risk PESI Vs 3.5% (761/21621) in patients with high-risk PESI. The summary OR of patients with low-risk PESI compared with patients with high-risk PESI was 0.34 (95% CI 0.29 to 0.41; Z=12.32, P < 0.00001) (Figure 5). The funnel plot showed no asymmetry, which indicated no evidence of publication bias (Figure 3C). While in subgroup analysis for sPESI and PESI, the pooled serious adverse events rate was 1.3% (85/6553) in patients with low-risk sPESI Vs 2.9% (377/11605) in patients with high-risk sPESI, the summary OR of patients with low-risk sPESI compared with patients with high-risk sPESI was 0.40 (95% CI 0.31 to 0.51; Z=7.59, P < 0.00001) (Figure 5 Above); the pooled serious adverse events rate was 1.2% (81/6802) in patients with low-risk PESI Vs 3.8% (384/10016) in patients with high-risk PESI, the summary OR of patients with low-risk PESI compared with patients with high-risk PESI was 0.30 (95% CI 0.23 to 0.38; Z=9.81, P < 0.00001) (Figure 5 Below).

**Figure 6:**
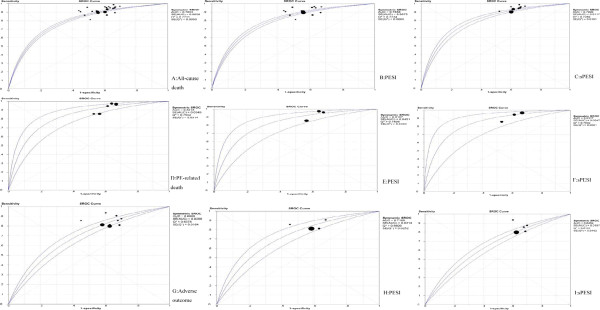
Sensitivity analysis of all-cause mortality (primary outcome) by removal the retrospective studies.

Sensitivity analysis of primary outcome (all-cause mortality) by removal the retrospective studies showed there was no heterogeneity in the studies (I^2^ = 1%); the summary OR of patients with low-risk PESI compared with patients with high-risk PESI was 0.13 (95% CI 0.10 to 0.19; Z=11.81, P < 0.00001) (Figure 6); the funnel plot showed no asymmetry, which indicated no evidence of publication bias (Figure 3D). Sensitivity analyses showed the robust result of the pooled meta-analyses.

#### Accuracy of the PESI to predict prognostic outcomes

The sensitivity and specificity for PESI in PE prognosis diagnosis of each included studies are also shown in Table 2.

**Figure 7:**
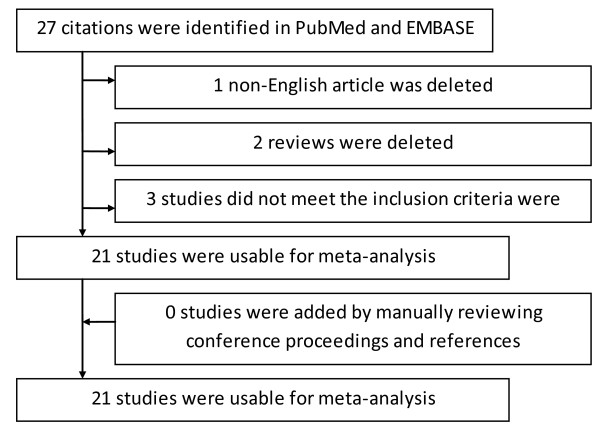
SROC plots for combined sensitivity and (1-specificity) of the included studies.

For the outcome of all-cause mortality, the pooled sensitivity was 0.909 (95% CI: 0.900 to 0.916). The pooled specificity was 0.411 (95% CI: 0.407 to 0.415). The SROC curve is shown in Figure 7A. The overall weighted AUC was 0.7853±0.0058, and the overall accuracy (Q*) was 0.7231±0.0050. While in the PESI subgroup, the pooled sensitivity was 0.900 (95% CI: 0.889 to 0.911), the pooled specificity was 0.411 (95% CI: 0.407 to 0.415), the SROC curve is shown in Figure 7B, the overall weighted AUC was 0.7856±0.0075, and the overall accuracy (Q*) was 0.7234±0.0065; in the sPESI subgroup, the pooled sensitivity was 0.919 (95% CI: 0.907 to 0.930), the pooled specificity was 0.382 (95% CI: 0.376 to 0.388), the SROC curve is shown in Figure 7C, the overall weighted AUC was 0.7920±0.0117, and the overall accuracy (Q*) was 0.7289±0.0101.

For the outcome of PE-related mortality, the pooled sensitivity was 0.953 (95% CI: 0.913 to 0.978). The pooled specificity was 0.374 (95% CI: 0.360 to 0.388). The SROC curve is shown in Figure 7D. The overall weighted AUC was 0.8218±0.0349, and the overall accuracy (Q*) was 0.7552±0.0314. While in the PESI subgroup, the pooled sensitivity was 0.949 (95% CI: 0.892 to 0.981), the pooled specificity was 0.382 (95% CI: 0.362 to 0.401), the SROC curve is shown in Figure 7E, the overall weighted AUC was 0.8158±0.0451, and the overall accuracy (Q*) was 0.7498±0.0403; in the sPESI subgroup, the pooled sensitivity was 0.960 (95% CI: 0.888 to 0.992), the pooled specificity was 0.365 (95% CI: 0.345 to 0.386), the SROC curve is shown in Figure 7F, the overall weighted AUC was 0.8317±0.0547, and the overall accuracy (Q*) was 0.7642±0.0501.

For the outcome of serious adverse events, the pooled sensitivity was 0.821 (95% CI: 0.795 to 0.845). The pooled specificity was 0.389 (95% CI: 0.384 to 0.394). The SROC curve is shown in Figure 7G. The overall weighted AUC was 0.6809±0.0208, and the overall accuracy (Q*) was 0.6378±0.0164. While in the PESI subgroup, the pooled sensitivity was 0.826 (95% CI: 0.788 to 0.859), the pooled specificity was 0.415 (95% CI: 0.407 to 0.422), the SROC curve is shown in Figure 7H, the overall weighted AUC was 0.7100±0.0314, and the overall accuracy (Q*) was 0.6609±0.0252; in the sPESI subgroup, the pooled sensitivity was 0.816 (95% CI: 0.778 to 0.850), the pooled specificity was 0.366 (95% CI: 0.358 to 0.373), the SROC curve is shown in Figure 7I, the overall weighted AUC was 0.6454±0.0197, and the overall accuracy (Q*) was 0.6101±0.0152.

## Discussion

Some meta-analysis and systematic reviews have provided sufficient evidence that the prognostic outcomes of APE can be affected by haemodynamic status, right ventricular dysfunction, myocardial injury, and other clinical features [37-41]. PESI was designed based on some clinical features. Our meta-analysis showed high-risk PESI is a risk factor for all-cause death, PE-related death, and serious adverse events. Pooled sensitivity, specificity, and SROC were used for predicting PE prognosis. An AUC value of 0.5 indicates that the test has no discriminatory ability, whereas an AUC value of 1.0 indicates perfect capability [42]. While to demonstrate excellent accuracy, the AUC should be in the region of 0.97 or above; an AUC of 0.93 to 0.96 is very good; 0.75 to 0.92 is good; but an AUC less than 0.75 has obvious deficiencies in its accuracy [43]. Our meta-analysis showed the overall weighted AUC for all-cause mortality was 0.78 (95% CI: 0.77 to 0.80); and AUC for PE-related mortality was 0.82 (95% CI: 0.75 to 0.89). PESI has good accuracy for predicting PE prognosis. The main reason is that the PESI was derived form multi-center large sample studies with appropriate research methods. In the PESI study, 15,531 discharged patients with PE treated at 186 hospitals were included, on the basis of the β-coefficients of the stepwise logistic regression model, PESI with a point score was generated [9].

Our meta-analysis showed in sPESI subgroup, the OR of all-cause mortality, PE-related mortality, and serious adverse events was similar to that in PESI subgroup. Most important, in sPESI subgroup, the AUC for predicting all-cause mortality, PE-related mortality, and serious adverse events was also similar to that in PESI subgroup. It has the same accuracy between PESI and sPESI. The sPESI is also derived form logistic regression analysis [10]. In the derivation data set, univariate logistic regression of the original 11 PESI variables led to the removal of variables that did not reach statistical signifi-cance and subsequently produced the simplified PESI that contained the variables of cancer, chronic cardiopulmonary disease, heart rate, systolic blood pressure, and oxyhemoglobin saturation levels. 11 variables in PESI reduced to 6 variables in sPESI. The sPESI does not decrease prognostic accuracy compared with the original PESI, but sPESI is easier to use.

In general, the performance of prognostic models should be assessed in two ways: its discrimination and calibration aspects. Discrimination is the ability of the model to correctly separate the subjects into different groups. Calibration is the degree of correspondence between the estimated probability produced by the model and the actual observed probability [6]. Discrimination is also called predicting accuracy, which can be assessed by sensitivity (true positive rate), and specificity (true negative rate), ROC, and AUC. Our meta-analysis has already proved the PESI has good accuracy for predicting APE outcomes. The calibration is also assessed by the Hosmer-Lemeshow test [44]. But the data of the calibration was not found in the included studies. So we can’t calculate the calibration for predicting prognosis in this meta-analysis. Some prospective studies for accessing PESI predicting calibration can be recommended.

Except we can’t calculated the calibration for predicting prognosis in this meta-analysis, there are some other limitations. First, Lankeit M maybe used same participants in two included studies [17] and [19], as well as Singanayagam A’s studies [26,29], and Jiménez D’s studies [34,35]. Repeated include will increase the weight of the studies, and maybe result bias. Second, different PE haemodynamic status of patients included in our meta-analysis, some included haemodynamic stable patients, some included haemodynamic stable and unstable patients. But this bias is small, because evaluation of hemodynamic status is included in the PESI and sPESI. Third, the meta-analysis can’t test the prognostic accuracy against other clinical score such as the Geneva prognostic score. Finally, most of the included studies were came from Europe and North America, only a study came from Asia, no study came from Africa, South America and Oceania. Some high-quality studies should be performed in these areas before PESI used worldwide.

## Conclusions

Our meta-analysis identified PESI has discriminative power to predict the short-term death and adverse outcome events in patients with acute pulmonary embolism; the PESI and the sPESI have similar accuracy, while sPESI is easier to use. However, the calibration for predicting prognosis can’t be calculated from this meta-analysis, some prospective studies for accessing PESI predicting calibration can be recommended.

## Competing interests

The authors declare that they have no competing interests.

## Authors’ contributions

XYZ and HLC reviewed the full text articles, extracted data and wrote the initial draft of the manuscript. SQB conducted the literature search, reviewed titles, abstracts and full text articles and contributed to the extraction of data. HLC reviewed titles and abstracts and provided critical revision of the manuscript. SSN and HLC contributed to the critical revision of the manuscript. All authors read and approved the final manuscript.

## Supplementary Material

Additional file 1 Pulmonary Embolism Severity Index (PESI), Simplified PESI (sPESI). Click here for file

Additional file 2 Newcastle - Ottawa Quality Assessment Scale. Click here for file
