# Supplementary material for: The prognostic value of pulmonary embolism severity index in acute pulmonary embolism: a meta-analysis
Source: Respir Res. 2012 Dec 4;13(1):111. doi: 10.1186/1465-9921-13-111 (PMC3571977; doi:10.1186/1465-9921-13-111)
Supplement: Additional file 1 — Pulmonary Embolism Severity Index (PESI), Simplified PESI (sPESI). [file 1465-9921-13-111-S1.doc]

**Pulmonary Embolism Severity Index (PESI**)

| Predictors | Points  assigned |
| --- | --- |
| Age | Age in years |
| Male sex | +10 |
| Cancer | +30 |
| Heart failure | +10 |
| Chronic lung disease | +10 |
| Pulse ≥ 110 /min | +20 |
| Systolic blood pressure < 100 mmHg | +30 |
| Respiratory rate ≥ 30/min | +20 |
| Temperature < 36°C/ 96.8° F | +20 |
| Altered mental status  (Disorientation, lethargy, stupor, or coma) | +60 |
| O_2_ Saturation < 90% on Room Air? | +20 |

Risk Class I (Very low): Points≤ 65; Risk Class II (Low): Points 66-85; Risk Class III (Intermediate): Points 86-105; Risk Class IV (High): Points 106- 125; Risk Class V (Very high): Points ≥ 126

Risk Class Ⅰ-Ⅱ(Low): Points≤85; Risk Class Ⅲ-Ⅴ(High): Points＞85

**Simplified PESI (sPESI)**

| Predictors | Points  assigned |
| --- | --- |
| Age > 80 years | +1 |
| Cancer | +1 |
| Chronic cardiopulmonary disease | +1 |
| Pulse ≥ 110 /min | +1 |
| Systolic blood pressure < 100 mmHg | +1 |
| Arterial blood oxygen saturation | +1 |

Risk Class Low: Points=0; Risk Class High: Points≥1
